# Patient Outcomes With Dose Escalation Using Modern Radiotherapy Techniques: A Retrospective Review of Anal Cancer Treated at a Large Academic Institution Between 2010 and 2016

**DOI:** 10.7759/cureus.10989

**Published:** 2020-10-16

**Authors:** Sonja C Murchison, Kimberly J DeVries, Siavash Atrchian

**Affiliations:** 1 Radiation Oncology, BC Cancer - Victoria, Victoria, CAN; 2 Population Oncology, Cancer Surveillance & Outcomes, BC Cancer - Vancouver, Vancouver, CAN; 3 Radiation Oncology, BC Cancer - Kelowna, Kelowna, CAN

**Keywords:** anal canal cancer, radiotherapy (rt), modern radiotherapy techniques, dose escalation

## Abstract

Introduction: The use of modern radiotherapy techniques (MRTs) has contributed to reduced treatment-related toxicities through better avoidance of normal structures and dose tapering, and has enabled the delivery of higher doses continuously. The purpose of this study was to review retrospectively (1) outcomes for anal cancer treated at BC Cancer (Canada) using MRT, and (2) the utilization and effect of dose escalation on cancer-related outcomes.

Methods: Patients between 2010 and 2016 with biopsy-proven anal cancer, aged >18 years, and treated with primary curative-intent chemoradiation using intensity modulated radiotherapy (IMRT) or volumetric modulated arc therapy (VMAT) were included. Primary end points included overall survival (OS), relapse-free survival (RFS), and colostomy-free survival (CFS). Kaplan-Meier curves were created for prognostic factors, as well as dose escalation (>54 Gy vs. ≤54 Gy). Univariate and multivariate analyses were performed to evaluate predictors of the outcome.

Results: A total of 273 patients were assessed. The median age was 61 years with 70% being female, 6% HIV positive, and 68% with locally advanced cancer (T3-4, or node positive). The median follow-up time was 41.3 months. Time from diagnosis to treatment was 60 days, and treatment duration 42 days. Dose escalation was prescribed for 22, of whom 15 were locally advanced cases. A total of 97% completed their radiation, including all who were dose-escalated; 11% required unplanned treatment breaks, with over half of breaks <5 days. More than 90% completed at least half of their chemotherapy; 41% had pre-treatment, and 34% post-treatment positron emission tomography (PET) scans. For primary tumor response, 88% were complete and 10% partial; 23% relapsed, with 15% locoregional, 5% distant, and 3% both, and 12% had salvage surgery. The colostomy rate was 15%, with 4% pre-treatment, 10% relapse related, and only 1% treatment-toxicity related. On univariate analysis, male sex was associated with a higher risk of death (p=0.02) and relapse (p=0.041). Non-squamous histology was consistently a strong predictor of all outcomes (OS, p=0.0089; RFS, p<0.0001; CFS, p<0.0001) as was advanced T stage (OS, p=0.0075; RFS, p=0.0019; CFS, p=0.0099), and node positivity (OS, p=0.0014; RFS, p=0.001; CFS, p=0.0071). Age, HIV status, grade, longer treatment times (>42-day median), and lack of a pre- or post-treatment PET scan were not associated with the outcome. Dose escalation beyond 54 Gy was not significant, even among locally advanced tumors. On multivariate analysis, non-squamous histology (OS, p=0.043; RFS, p<0.001; CFS, p=0.01), T4 (OS, p=0.049; RFS, p=0.026; CFS, p=0.042) and node positivity (OS, p=0.05; RFS, p=0.006) remained significant predictors of the outcome, although node positivity was no longer significant for CFS (p=0.10).

Conclusion: BC Cancer outcomes for anal cancer treated with MRTs are comparable to what has been previously reported. Unplanned breaks were notably few, and short. Treatment-related colostomies were rare. Dose-escalated regimens were infrequently prescribed, appeared tolerable, but more often required a break. Prospective trials are needed to clarify efficacy of such regimens.

## Introduction

It has long been established that combined modality treatment of anal cancer using chemo-radiotherapy (CRT) results in superior outcomes [1,2]. Although this is the standard of care, locally advanced cases still do poorly. As per the American Joint Committee on Cancer (AJCC) Cancer Staging Manual, Eighth Edition [3], stages I-IIA (T1-2, N0) have five-year relapse-free survival (RFS) and overall survival (OS) rates approaching 80%-85%, compared to more advanced stages (T3-4, or N1), where OS and RFS may be as low as 40%.

The value of dose escalation for the treatment of anal cancer, whether early stage or locally advanced, is uncertain, but has been previously explored as a way to improve the outcomes for anal cancer. Because of the toxicities associated with CRT, older studies often prescribed a break partway through treatment [4,5], sometimes amounting to an interruption of several weeks. For example, Radiation Therapy Oncology Group (RTOG) 92-08, a phase II trial of dose escalation, mandated a two-week treatment break to mitigate the substantial acute toxicity [4]. Subsequent studies have shown breaks to be detrimental [1,4-6]. It is worth noting that many of these earlier studies establishing CRT as a standard of care employed older non-conformal radiation techniques [1,2,4,5]. Newer radiation techniques, which allow dose painting and better avoidance of normal structures, have been shown to reduce these treatment-related toxicities, allowing for the delivery of higher doses in a continuous manner [7-17].

Recent studies using modern radiotherapy techniques (MRTs) such as intensity modulated radiation therapy (IMRT) or volumetric modulated arc therapy (VMAT) have been fairly small [8,11-14,18-19], which reflects the fact that anal cancer is relatively uncommon, with about a 100 new cases annually in British Columbia, and about 500 Canada-wide [20]. At BC Cancer (BCC), anal cancer patients since 2010 have received uninterrupted concurrent CRT, using MRTs. As the sole institution providing cancer treatment on a province-wide basis, the larger number of patients seen at this institution would allow for a comprehensive, contemporary assessment of this disease and its treatment. The purpose of this study was to review retrospectively (1) outcomes for anal cancer treated with curative intent at BCC using MRTs, and (2) the utilization and effect of dose escalation on cancer-related outcomes.

## Materials and methods

Patients

This study included patients treated at an institution that provides all radiotherapy services provincially, and was approved by the institutional research ethics board. Between 2010 and 2016, all patients with pathologically proven anal carcinoma, including those with regional nodal involvement, above 18 years of age, treated at BCC using MRT were reviewed retrospectively. Patients were excluded if they had incomplete staging information, distant metastases from either anal cancer or another cancer primary, and if they were not treated with primary curative-intent therapy. The study period was chosen based on BCC’s adoption of MRT in 2010, and to allow adequate follow-up time (minimum two years) to observe the primary end point of RFS. For radiotherapy, patients underwent CT simulation, and gross tumor and elective nodal regions were contoured with margin expansions to create target volume. Chemotherapy delivered concurrently with radiotherapy most often included intravenous Mitomycin C (10 mg/m^2^ on day 1, weeks 1 and 5) and either 5-flurouracil (1000 mg/m^2^ for four days, weeks 1 and 5) or oral capecitabine (825 mg/m^2^ twice daily each radiation day), the latter being common practice at BCC due to ease of administration and tolerability.

Data collection

Clinical data was collected from the institution’s electronic medical record system, Cancer Agency Information System (CAIS), where all patients received follow-up. Data was collected for previously identified prognostic factors including patient age, gender, HIV infection status, tumor histology (squamous versus non-squamous), grade, T and N category [3]. Treatment information collected included radiotherapy dose and technique, chemotherapy regimen, treatment duration (days), and number, duration, and cause of unplanned treatment breaks. For clinical outcomes, tumor response as assessed by imaging and physical exam was collected. No evidence of residual tumor was considered a complete response (CR). Tumors unchanged or increased in size were considered stable and progressive disease, respectively. Colostomies were classified as pre-treatment (done before radiotherapy commenced due to the presence of tumor), disease related (done after radiotherapy in the presence of persistent or recurrent local disease), or toxicity related (done after radiotherapy in the absence of persistent or recurrent local disease).

Statistical analysis

Statistical analysis was performed using SAS Version 9.4 for Microsoft Windows (SAS Institute Inc., Cary, NC). The primary end points were RFS, defined as the time from end of treatment to recurrence (local or distant) or death from any cause, and OS, from the end of treatment until death from any cause. Colostomy-free survival (CFS) was defined as the time from end of treatment until colostomy surgery or death from any cause, and included pre-treatment colostomies not reversed by six months post-treatment.

The Kaplan-Meier method was used to estimate rates of RFS, OS, and CFS for the group. The log-rank test, two-tailed, with p<0.05 denoting significance, was used to assess differences in survival between groups. For RFS, patients were censored at the time of last exam or imaging showing stability if they had not progressed at the time of analysis. For OS and CFS, patients were censored at their last clinical encounter (i.e., any test, or visit confirming they were alive or colostomy-free, respectively).

Multivariate Cox proportional hazards analysis with adjustment for age and sex was performed to identify independent predictors of outcome. Variables included age, gender, HIV infection status, histologic subtype (squamous vs. non-squamous), grade, T category, N category, radiotherapy dose (≤54 Gy or >54 Gy to gross disease), and treatment duration (more than, or less than the median time).

## Results

The median follow-up time was 41.3 months. Of the 283 patients identified, 10 were excluded due to other metastatic cancer, incomplete staging, and receipt of nonstandard treatment regimens. Patient demographics and treatment information are included in Tables 1-2. The median prescribed dose was 54 Gy, ranging from 50 to 60 Gy. There were two treatment-related colostomies in patients prescribed 50.4 Gy to their primary tumor: one had a maximum point dose of 47.8 Gy and V45 Gy of 42 cc, and the other a maximum point dose of 53.75 Gy and V45 Gy of 65 cc. All patients prescribed dose-escalated regimens were able to complete their treatment, with 22.7% needing a break versus 10% of those receiving a standard dose. More than half of all breaks were five days or less, and three-quarters were less than 10 days.

**Table 1:** Patient demographics and treatments MMC, Mitomycin C; 5FU, 5-fluorouracil; RT, radiotherapy; IMRT, intensity modulated radiotherapy; VMAT, volumetric modulated arc therapy; PET, positron emission tomography

	Number	Percent	Total
Age (years)			
Median	61		
SD	9.9		
Sex			273
Male	82	30%	
Female	191	70%	
HIV			273
Positive	16	6%	
Negative	257	94%	
Histology			273
Squamous	266	97%	
Non-squamous	7	3%	
Differentiation			273
Well	29	11%	
Moderate	113	41%	
Poor	53	19%	
Unknown	78	29%	
HPV			273
Positive	96	35%	
Negative	3	1%	
Unknown	174	64%	
T stage			273
1	39	14%	
2	100	37%	
3	90	33%	
4	44	16%	
N stage			273
0	125	46%	
1	60	22%	
2	49	18%	
3	39	14%	
Locally advanced (T3-4, N+)			
No	86	68%	273
Yes	187	32%	
Chemotherapy regimen			273
MMC and capecitabine	196	72%	
MMC and 5FU	51	19%	
Capecitabine only	8	3%	
Cisplatin and capecitabine	9	3%	
Cisplatin and 5FU	5	2%	
None	4	1%	
Chemotherapy completed			269
Yes	198	74%	
No, more than half	50	19%	
No, less than half	21	8%	
RT technique			273
IMRT	111	41%	
VMAT	162	59%	
RT dose prescribed			273
≤54 Gy	251	92%	
>54 Gy	22	8%	
Range	50–60 Gy		
RT complete			273
Yes	266	97%	
No	7	3%	
Unplanned RT break (days)			273
Yes	31	11%	
No	242	89%	
Median	5		
Range	1–69		
RT duration (days)			
Median	42		
Range	21–99		
Pre-treatment PET done			273
Yes	113	41%	
No	160	59%	
Post-treatment PET done			273
Yes	92	34%	
No	181	66%	
Median no. of days post-treatment	92.5		
SD	46		
Clear	53	58%	
Uncertain	25	27%	
Persistent disease	12	13%	
Time from diagnosis to treatment (days)			
Median	61		
SD	27		

**Table 2:** Treatment outcomes in patients

	Number	Percent	Total
Primary tumor response			273
Complete	240	88%	
Partial	26	10%	
Stable or progression	3	1%	
Unknown	4	1%	
Failure type			273
No failure	210	77%	
Locoregional	40	15%	
Distant	13	5%	
Both	10	4%	
Salvage type			32
Abdominoperineal resection	26	81%	
Pelvic exenteration	5	16%	
Metastatectomy and/or lymph node dissection	1	3%	
Colostomies			273
No	232	85%	
Pre-treatment	12	4%	
Treatment failure	27	10%	
Treatment complications	2	1%	

Overall, there were 63 (23.1%) relapses, 41 (15.0%) colostomies, and 55 (20.2%) all-cause deaths out of the total 273 patients in the sample. Kaplan-Meier estimates for one- and three-year survivals are provided in Tables 3-5. Of the 40 patients presenting with locoregional relapses, all but one (vulvar recurrence) were within the irradiated field. Among these patients, 32 proceeded with salvage surgery, and the remaining 8 were either unsuitable for salvage (comorbidities, extensive recurrence) or refused surgery.

**Table 3:** Overall survival in patients RT, radiotherapy; PET, positron emission tomography

		95% CI			95% CI	
	1-Year	Lower	Upper	3-Year	Lower	Upper
All	92.7%	88.8%	95.2%	80.7%	75.1%	85.3%
Age (years)						
<61	93.3%	87.5%	96.5%	82.2%	73.8%	88.1%
≥61	92.0%	86.1%	95.5%	79.4%	71.0%	85.6%
Sex						
Male	86.5%	77.0%	92.3%	73.4%	61.5%	82.1%
Female	95.3%	91.1%	97.5%	83.7%	77.0%	88.7%
HIV						
Negative	93.4%	89.5%	95.8%	81.5%	75.6%	86.1%
Positive	81.3%	52.5%	93.5%	68.8%	40.5%	85.6%
Histology						
Non-squamous	85.7%	33.4%	97.9%	57.1%	17.2%	83.7%
Squamous	92.8%	89.0%	95.4%	81.4%	75.7%	85.9%
Differentiation						
Unknown	97.4%	90.1%	99.4%	87.6%	77.4%	93.4%
Well	82.8%	63.4%	92.4%	79.3%	59.6%	90.1%
Moderate	91.1%	84.1%	95.1%	76.8%	66.8%	84.1%
Poor	94.2%	83.2%	98.1%	79.7%	64.2%	89.0%
T stage						
1	100.0%			91.5%	70.0%	97.8%
2	98.0%	92.2%	99.5%	85.4%	75.4%	91.6%
3	90.0%	81.7%	94.7%	76.8%	66.3%	84.4%
4	79.5%	64.4%	88.8%	69.1%	52.6%	80.9%
N stage						
0	96.0%	90.6%	98.3%	87.0%	78.3%	92.4%
1	90.0%	79.1%	95.4%	78.7%	65.4%	87.4%
2	93.9%	82.2%	98.0%	75.8%	60.3%	85.9%
3	84.6%	68.9%	92.8%	70.4%	52.8%	82.5%
RT dose						
≤54 Gy	93.6%	89.8%	96.0%	81.5%	75.6%	86.2%
>54 Gy	81.8%	58.5%	92.8%	71.8%	47.4%	86.3%
Treatment time (days)						
<42	88.3%	78.8%	93.8%	78.4%	66.4%	86.5%
≥42	94.4%	90.0%	96.8%	81.9%	75.2%	86.9%
Pre-treatment PET						
No	91.9%	86.4%	95.2%	81.4%	73.8%	86.9%
Yes	93.8%	87.4%	97.0%	80.1%	70.7%	86.8%
Post-treatment PET						
No	91.7%	86.6%	94.9%	80.3%	73.1%	85.7%
Yes	94.5%	87.3%	97.7%	81.8%	71.4%	88.7%
Locally advanced						
No	98.8%	92.0%	99.8%	85.9%	74.2%	92.5%
Yes	89.8%	84.5%	93.4%	78.4%	71.5%	83.9%
RT dose, for locally advanced (T3-4 and/or N+)						
≤54 Gy	91.3%	86.0%	94.6%	79.7%	72.5%	85.1%
>54 Gy	73.3%	43.6%	89.1%	64.2%	33.3%	83.6%

**Table 4:** Relapse-free survival in patients RT, radiotherapy; PET, positron emission tomography

		95% CI			95% CI	
	1-Year	Lower	Upper	3-Year	Lower	Upper
All	83.2%	78.2%	87.2%	76.0%	70.1%	80.9%
Age						
<61	82.7%	75.1%	88.2%	75.0%	66.4%	81.7%
≥61	83.7%	76.4%	89.0%	77.1%	68.5%	83.6%
Sex						
Male	75.1%	64.1%	83.2%	68.5%	56.4%	77.9%
Female	86.7%	81.0%	90.8%	79.2%	72.3%	84.5%
HIV						
Negative	83.8%	78.6%	87.8%	76.6%	70.6%	81.6%
Positive	75.0%	46.3%	89.8%	66.7%	36.9%	84.8%
Histology						
Non-squamous	28.6%	4.1%	61.2%	14.3%	0.7%	46.5%
Squamous	84.7%	79.7%	88.5%	77.7%	71.8%	82.5%
Differentiation						
Unknown	83.3%	73.0%	89.9%	74.4%	62.7%	83.0%
Well	82.0%	62.0%	92.1%	74.2%	53.2%	86.8%
Moderate	80.0%	71.3%	86.4%	72.8%	62.7%	80.6%
Poor	90.5%	78.6%	95.9%	86.2%	73.2%	93.2%
T stage						
1	94.8%	80.8%	98.7%	90.9%	73.6%	97.0%
2	89.9%	82.0%	94.4%	81.8%	72.3%	88.4%
3	77.0%	66.6%	84.5%	72.4%	61.2%	80.9%
4	70.1%	54.1%	81.4%	56.5%	39.9%	70.1%
N stage						
0	94.3%	88.4%	97.2%	87.3%	79.4%	92.4%
1	75.7%	62.4%	84.9%	67.7%	53.5%	78.4%
2	71.3%	56.4%	81.9%	68.3%	52.8%	79.7%
3	74.2%	57.3%	85.2%	63.0%	45.5%	76.2%
RT dose						
≤54 Gy	83.8%	78.6%	87.8%	75.9%	69.7%	81.0%
>54 Gy	77.3%	53.7%	89.8%	77.3%	53.7%	89.8%
Treatment time (days)						
<42	80.2%	69.3%	87.6%	74.0%	62.3%	82.7%
≥42	84.4%	78.5%	88.9%	76.7%	69.6%	82.3%
Pre-treatment PET						
No	85.3%	78.7%	90.0%	78.2%	70.4%	84.1%
Yes	80.4%	71.7%	86.6%	73.0%	63.4%	80.5%
Post-treatment PET						
No	88.2%	82.5%	92.1%	79.2%	71.9%	84.7%
Yes	73.7%	63.4%	81.5%	70.1%	59.5%	78.5%
Locally advanced						
No	96.5%	89.4%	98.8%	89.1%	79.0%	94.5%
Yes	77.1%	70.3%	82.5%	70.0%	62.5%	76.2%
RT dose, for locally advanced (T3-4 and/or N+)						
≤54 Gy	77.4%	70.3%	83.0%	69.7%	61.8%	76.2%
>54 Gy	73.3%	43.6%	89.1%	73.3%	43.6%	89.1%

**Table 5:** Colostomy-free survival in patients RT, radiotherapy; PET, positron emission tomography

		95% CI			95% CI	
	1-Year	Lower	Upper	3-Year	Lower	Upper
All	89.1%	84.6%	92.3%	84.3%	79.2%	88.3%
Age						
<61	82.7%	75.1%	88.2%	75.0%	66.4%	81.7%
≥61	83.7%	76.4%	89.0%	77.1%	68.5%	83.6%
Sex						
Male	86.0%	76.1%	92.0%	86.0%	76.1%	92.0%
Female	90.3%	85.1%	93.8%	83.8%	77.5%	88.5%
HIV						
Negative	89.2%	84.6%	92.5%	84.2%	78.9%	88.3%
Positive	87.5%	58.6%	96.7%	87.5%	58.6%	96.7%
Histology						
Non-squamous	34.3%	4.8%	68.5%	34.3%	4.8%	68.5%
Squamous	90.4%	86.1%	93.4%	85.5%	80.5%	89.3%
Differentiation						
Unknown	88.2%	78.5%	93.7%	85.2%	74.8%	91.5%
Well	92.7%	73.7%	98.1%	84.2%	63.0%	93.8%
Moderate	88.9%	81.3%	93.6%	82.6%	73.7%	88.7%
Poor	88.6%	76.4%	94.7%	86.4%	73.5%	93.3%
T stage						
1	97.4%	82.8%	99.6%	94.6%	80.0%	98.6%
2	93.9%	86.8%	97.2%	88.4%	80.0%	93.4%
3	86.1%	76.8%	91.9%	82.0%	71.8%	88.8%
4	76.3%	60.4%	86.6%	69.7%	52.3%	81.8%
N stage						
0	95.0%	89.2%	97.7%	90.5%	83.5%	94.6%
1	86.0%	73.9%	92.7%	79.8%	66.4%	88.3%
2	77.4%	62.9%	86.8%	74.9%	59.9%	84.9%
3	89.3%	73.9%	95.9%	82.4%	64.6%	91.8%
RT dose						
≤54 Gy	90.1%	85.6%	93.3%	85.0%	79.7%	89.0%
>54 Gy	76.7%	52.7%	89.6%	76.7%	52.7%	89.6%
Treatment time (days)						
<42	87.5%	77.3%	93.3%	79.7%	68.1%	87.5%
≥42	89.6%	84.3%	93.1%	86.0%	80.1%	90.3%
Pre-treatment PET						
No	88.9%	82.8%	93.0%	83.7%	76.7%	88.8%
Yes	89.2%	81.7%	93.7%	85.1%	76.8%	90.6%
Post-treatment PET						
No	91.3%	86.0%	94.7%	86.1%	79.8%	90.6%
Yes	84.6%	75.4%	90.6%	80.9%	71.0%	87.7%
Locally advanced						
No	96.4%	89.3%	98.8%	92.7%	84.4%	96.6%
Yes	85.6%	79.6%	90.0%	80.4%	73.5%	85.6%
RT dose, for locally advanced (T3-4 and/or N+)						
≤54 Gy	86.7%	80.5%	91.0%	81.0%	73.9%	86.4%
>54 Gy	73.3%	43.6%	89.1%	73.3%	43.6%	89.1%

**Figure 1:**
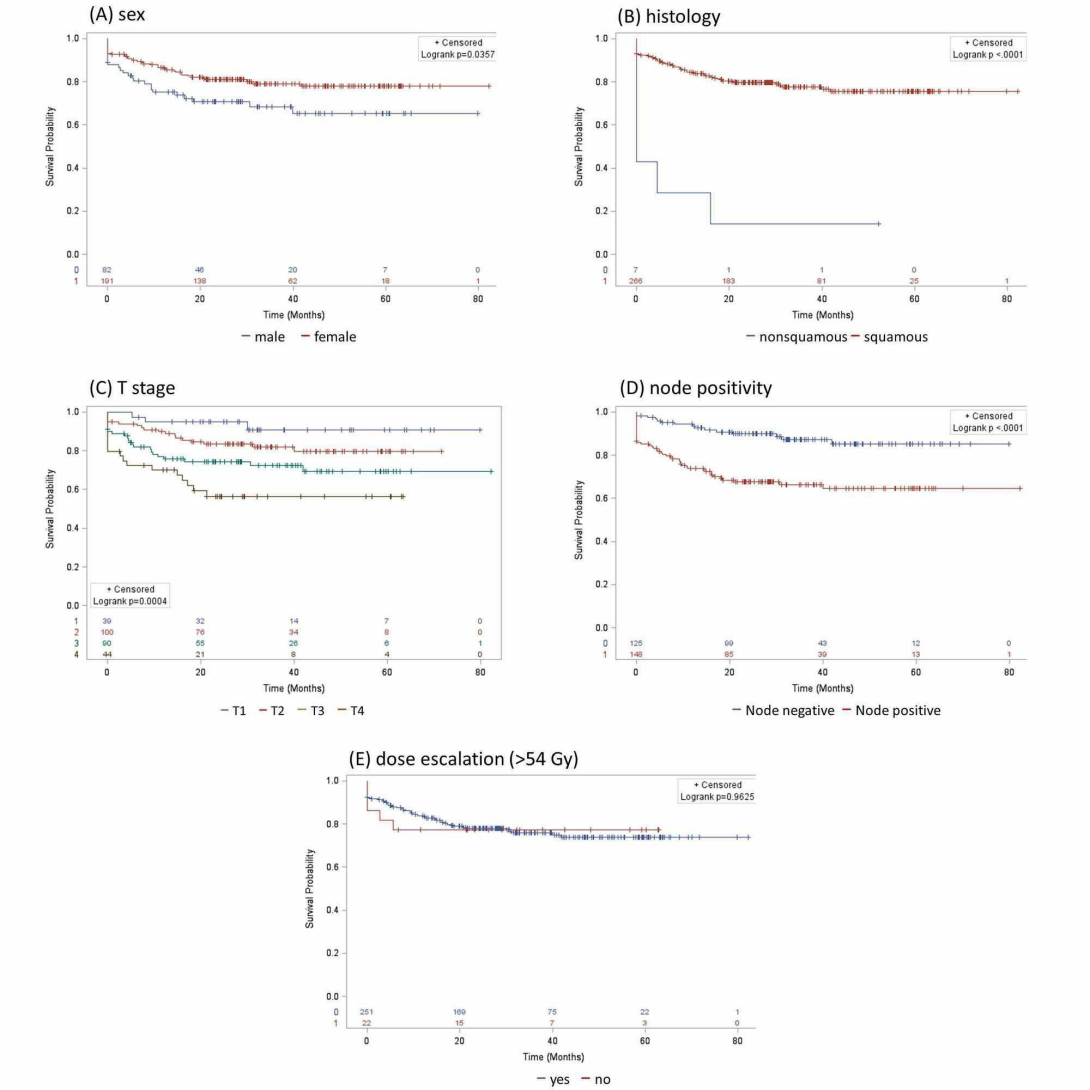
Univariate analysis showed a significant association between relapse-free survival and sex (A), histology (B), T stage (C), and node positivity (D). Dose escalation beyond 54 Gy (E), age, HIV status, grade, and lack of a pre- or post-treatment PET scan were not significant. PET, positron emission tomography

On univariate analysis, male sex was associated with a higher risk of death (p=0.02) and relapse (p=0.041) (Table 6, Figure 1). Non-squamous histology was a consistently strong predictor of a higher risk of death (p=0.009), relapse (p<0.0001), and colostomy surgery (p<0.001). In general, higher T stage and N stage were associated with a higher risk of death (T3, p=0.024; T4, p=0.008; N1, p=0.01; N2, p=0.01; N3, p=0.005), relapse (T3, p=0.02; T4, p=0.002; N1, p=0.002; N2, p=0.004; N3, p<0.001), and colostomy (T4, p=0.01; N1, p=0.03; N2, p=0.006). Node positivity was a significant predictor of all outcomes (OS, p=0.001; RFS, p=0<0.001; CFS, p=0.007). Dose escalation beyond 54 Gy was not statistically significant, even when assessed separately for locally advanced disease (T3-4 and/or node positive). Age, HIV status, grade, lack of a pre- or post-treatment positron emission tomography (PET) scan were also not statistically significant.

**Table 6:** Univariate analysis data RT, radiotherapy; PET, positron emission tomography; OS, overall survival; RFS, relapse-free survival; CFS, colostomy-free survival; HR, hazard ratio; REF, reference

	OS			RFS			CFS		
	HR	95% CI	p value	HR	95% CI	p value	HR	95% CI	p value
Age (years)									
<61	REF			REF			REF		
≥61	1.19	0.70–2.02	0.5298	0.84	0.51–1.38	0.4852	0.7	0.37–1.29	0.2507
Sex									
Female	REF			REF			REF		
Male	1.89	1.10–3.23	0.0202	1.7	1.02–2.81	0.0406	0.9	0.45–1.79	0.7598
HIV									
No	REF			REF			REF		
Yes	2.12	0.91–4.96	0.0818	1.66	0.66–4.13	0.2803	0.93	0.22–3.84	0.9153
Squamous histology									
Yes	REF			REF			REF		
No	3.9	1.41–10.82	0.0089	7.05	3.01–16.53	<0.0001	5.84	2.07–16.50	0.0009
Grade									
1	REF			REF			REF		
2	0.75	0.34–1.66	0.4717	0.99	0.43–2.26	0.9723	1.15	0.39–3.40	0.8003
3	0.6	0.24–1.52	0.2824	0.48	0.17–1.37	0.1695	0.93	0.27–3.18	0.9095
Unknown	0.46	0.19–1.12	0.0858	1.03	0.44–2.42	0.9486	1.06	0.34–3.29	0.9171
T stage									
1	REF			REF			REF		
2	1.67	0.48–5.85	0.4244	2.44	0.72–8.29	0.1518	2.42	0.54–10.83	0.2464
3	3.97	1.20–13.15	0.0241	4.09	1.23–13.58	0.0215	3.83	0.88–16.75	0.0745
4	5.49	1.58–19.11	0.0075	6.97	2.05–23.67	0.0019	7.18	1.61–32.14	0.0099
N stage									
0	REF			REF			REF		
1	2.55	1.25–5.23	0.0105	2.94	1.48–5.85	0.0021	2.55	1.12–5.77	0.0253
2	2.59	1.24–5.44	0.0118	2.88	1.41–5.90	0.0038	3.16	1.39–7.16	0.0059
3	3.11	1.41–6.86	0.0049	3.83	1.87–7.84	0.0002	1.94	0.72–5.23	0.1936
RT dose									
≤54 Gy	REF			REF			REF		
>54 Gy	1.37	0.59–3.21	0.4637	1.02	0.41–2.55	0.9626	1.78	0.70–4.53	0.2295
RT dose for locally advanced									
≤54 Gy	REF			REF			REF		
>54 Gy	1.46	0.57–3.70	0.4311	0.9	0.32–2.48	0.8336	1.67	0.59–4.73	0.3359
Overall treatment time (days)									
<42	REF			REF			REF		
≥42	1.19	0.68–2.10	0.5412	1.33	0.79–2.24	0.2894	1.35	0.71–2.58	0.358
Pre-treatment PET									
No	REF			REF			REF		
Yes	1.2	0.70–2.05	0.5019	1.24	0.75–2.03	0.3975	0.99	0.53–1.85	0.9822
Post-treatment PET									
No	REF			REF			REF		
Yes	1.01	0.57–1.77	0.9794	1.59	0.97–2.62	0.0686	1.59	0.86–2.95	0.1392

On multivariate Cox proportional hazards analysis, non-squamous histology (OS, p=0.043; RFS, p<0.001; CFS, p=0.01), T stage 4 (OS, p=0.049; RFS, p=0.026; CFS, p=0.042), and node positivity (OS, p=0.05; RFS, p=0.006) continued to be associated with a higher risk of death, relapse, and colostomy surgery, although node positivity was no longer statistically significant in the multivariate analysis of colostomy-free survival (p=0.10) (Table 7).

**Table 7:** Multivariate Cox regression analysis data RT, radiotherapy; OS, overall survival; RFS, relapse-free survival; CFS, colostomy-free survival; HR, hazard ratio; REF, reference

	OS			RFS			CFS		
	HR	95% CI	p value	HR	95% CI	p value	HR	95% CI	p value
Age (years)									
<61	REF			REF			REF		
≥61	1.14	0.65–2.00	0.6378	0.85	0.50–1.44	0.547	0.63	0.33–1.19	0.1571
Sex									
Female	REF			REF			REF		
Male	1.56	0.88–2.76	0.1274	1.62	0.94–2.77	0.0813	0.79	0.38–1.65	0.5253
HIV									
No	REF			REF			REF		
Yes	1.44	0.56–3.68	0.4448	1.06	0.39–2.89	0.9034	0.69	0.15–3.10	0.6293
Squamous histology									
Yes	REF			REF			REF		
No	2.97	1.04–8.49	0.0426	6.02	2.42–14.95	0.0001	4.01	1.35–11.98	0.0127
T stage									
1	REF			REF			REF		
2	1.62	0.46–5.71	0.4533	2.57	0.75–8.78	0.1321	2.43	0.54–10.96	0.247
3	2.85	0.84–9.71	0.0945	3.21	0.94–10.95	0.0628	3.72	0.82–16.97	0.0895
4	3.65	1.01–13.23	0.0488	4.22	1.19–14.93	0.0256	5.04	1.06–24.03	0.0423
Node positive									
No	REF			REF			REF		
Yes	1.93	1.01–3.67	0.0461	2.39	1.29–4.46	0.0059	1.87	0.89–3.95	0.0997
RT dose									
≤54 Gy	REF			REF			REF		
>54 Gy	1.13	0.48–2.69	0.7802	0.88	0.35–2.22	0.7802	1.93	0.74–5.06	0.1786

## Discussion

This study assessing the use of MRTs for the treatment of anal cancer found that the majority of patients completed their prescribed treatment with few treatment breaks that were generally short. Prior studies have shown a clear advantage of MRTs over older conformal ones [12]. Other studies involving modern techniques like ours have shown unplanned treatment breaks to be infrequent and brief, with the percentage of patients requiring them broadly ranging from less than 10% to 35%. Therefore, at the lower end of that spectrum, our finding that 11% required an unplanned break is reassuring. Likewise, outcomes were comparable to those reported elsewhere. For early-stage disease, RFS and OS have both been reported at approximately 80%-85%, compared to our institution’s RFS of 89% and OS of 84%. For advanced-stage disease, a broader range for RFS (40%-80%) and OS (40%-70%) has been reported. Our results, with RFS at 67% and OS 72%, lie at the higher end of that range that is also encouraging. Our CFS rate (85%) and high rate of salvage (80%) for locoregional relapses is also similar to other studies [11]. The current study supports the idea that conformal techniques may be adopted while maintaining treatment efficacy. Furthermore, as a large institution providing care province-wide, the current data is useful for clinicians making treatment recommendations and counselling patients.

The lack of a pre- or post-treatment PET scan did not appear to affect outcomes, which is reassuring for places where access to this imaging tool may be limited. This does not, however, indicate that it is unimportant for treatment and management. PET scans are valuable for accurate staging, and for guiding management, including the delineation of radiation target volumes and prognostication [21]. It is conceivable that PET scans, given their usefulness as a prognostic tool, could enable early salvage before distant disease develops, but given that the number of patients in this scenario is small, such a benefit may be difficult to detect.

Very few colostomies (1%) were required for treatment-related toxicity. Although there is ample data on toxicity rates in general, less is known about cause-specific colostomy rates. One multicenter cohort study of 235 patients found the five-year rate of toxicity-related colostomies to be 8%; however, the median dose was much higher (64 Gy) using older radiation techniques most often without chemotherapy [22]. Contemporary studies comparing old and new techniques with a lower mean dose (53.5 Gy) have not shown a significant difference between techniques, with a low toxicity-related colostomy rate of 3% [23]. One recent study of IMRT with a prescribed dose of 54 Gy reported no treatment-related colostomies [13]. These findings from contemporary studies suggest that doses up to 54 Gy are quite safe and that dose escalation beyond 54 Gy, given the well-established improved side effect profile of MRT, is a reasonable question to explore. In particular, it is important to learn more about the potential late side effects of dose-escalated treatments that use modern techniques, since although the treatment may be more tolerable due to reduced acute side effects, fibrosis and problems contributing to sphincter dysfunction may still be problematic.

In this study, acknowledging that the number of evaluable cases was low, a higher dose was not associated with an improved outcome, even when assessed separately for locally advanced tumors. Other tumor sites showing dose-related response, including head and neck and gynecologic squamous cell cancers, often prescribe a dose higher than 59.4 Gy, so it is possible this dose is inadequate. An EQD2 of 70 Gy is routinely achieved for head and neck sites using MRT, and 60 Gy for cervix using a combination of MRTs and brachytherapy. Other established prognostic factors were significant [3], suggesting that our findings are applicable to other populations studied. From the data, dose-escalated regimens appeared tolerable since 100% of patients in this group completed their prescribed treatment. Compared to standard (<54Gy) regimens, 22.7% versus 10% required an unplanned treatment break. Others have reported low rates of treatment breaks for dose-escalated cases using MRTs. For example, Tomasoa et al. showed that 95% of 106 patients completed the planned treatment of dose-escalated RT to 59.4 Gy, of whom 6% required a treatment break [8]. In Franco et al.'s study, where some patients were prescribed up to 60 Gy (mean 54 Gy), 17% required an unplanned treatment break [9]. This suggests that factors other than the dose received and delivery technique contribute to the need for unplanned breaks, and also that it is possible for patients to complete a dose-escalated course without requiring one.

The strengths of this study are its large size, and a consistent use of MRTs such as IMRT and VMAT. To our knowledge, with 273 patients, this is the largest outcome study of anal cancer treated with MRTs [11-14,18-19], prior to which the largest involved 165 patients [12]. We report good survival outcomes that are comparable to other modern reports, and low rates of locoregional failure, for which the successful salvage rates are high. Treatment-related colostomy rates were exceptionally low, with none occurring in the dose-escalated group. This is encouraging, since in addition to assessing its benefit in cancer control, safety and preservation of organ function is a concern when investigating dose escalation.

Limitations of this study include the retrospective nature of the data, particularly, the inability to account for confounding variables, and relatively short follow-up, as median survival was not yet reached. Toxicity data was inconsistently available; however, treatment-related colostomies may provide some insight, and are a relevant end point to consider since a major advantage of CRT is organ preservation, which as discussed has been less well-studied than other treatment toxicities. Finally, few patients (22/273) were prescribed dose-escalated protocols, which in the absence of randomized prospective data, is understandable.

In the future, data from randomized controlled trials evaluating dose escalation, such as PersonaLising Anal cancer radioTherapy dOse (PLATO) [24], will prove valuable. From our study, dose-escalated protocols appear tolerable, but there may be a risk of requiring more breaks. A clear benefit was not shown, but it is acknowledged that the numbers receiving higher doses were low. It is reasonable to explore higher treatment doses if there exists the possibility of (1) better tumor control or cure and (2) organ preservation; it is also important that (3) CRT plus salvage does not result in worse outcomes than upfront surgery and (4) that there is a way to treat CRT-related toxicity. Although we do not know if dose escalation offers improved local control or cure, we do know that standard doses often fail in locally advanced patients; as such, these patients have a higher chance of requiring a colostomy, which warrants an attempt to improve the outcomes in this group.

## Conclusions

MRTs allow for good treatment outcomes, with extremely low rates of treatment-related colostomy. Locally advanced tumors still do poorly. This was not improved by dose escalation, although the number of cases in this study was low. Prospective studies are required to confirm the value of dose escalation in these patients.
